# Advances in Methane Emission Estimation in Livestock: A Review of Data Collection Methods, Model Development and the Role of AI Technologies

**DOI:** 10.3390/ani14030435

**Published:** 2024-01-29

**Authors:** Jalil Ghassemi Nejad, Mun-Su Ju, Jang-Hoon Jo, Kyung-Hwan Oh, Yoon-Seok Lee, Sung-Dae Lee, Eun-Joong Kim, Sanggun Roh, Hong-Gu Lee

**Affiliations:** 1 Department of Animal Science and Technology, Sanghuh College of Life Sciences, Konkuk University, Seoul 05029, Republic of Korea; jalilgh@konkuk.ac.kr (J.G.N.); ju2139@konkuk.ac.kr (M.-S.J.); godandthegod@konkuk.ac.kr (J.-H.J.); animal0502@konkuk.ac.kr (K.-H.O.); 2 School of Biotechnology, Hankyong National University, Anseong 17579, Republic of Korea; yoonseok95@hknu.ac.kr; 3 Center for Genetic Information, Hankyong National University, Anseong 17579, Republic of Korea; 4 Animal Nutrition and Physiology Division, National Institute of Animal Science, Rural Development Administration, Wanju 55365, Republic of Korea; leesd@korea.kr; 5 Department of Animal Science, Kyungpook National University, Sangju 37224, Republic of Korea; ejkim2011@knu.ac.kr; 6 Graduate School of Agricultural Science, Tohoku University, Sendai 980-8572, Japan; sangun.roh@tohoku.ac.jp

**Keywords:** methane emission estimation, model making and AI technologies

## Abstract

**Simple Summary:**

This paper explores the methane emissions from the livestock industry and their large impact on climate change, with a particular focus on cattle. It emphasizes how important it is to monitor and control methane accurately because it is a powerful greenhouse gas that accounts for 14–16% of world emissions. The study evaluates both conventional and AI-powered techniques for methane emission detection, emphasizing the significance of cattle in particular. It has been determined that region-specific formulations are required. The review discusses a number of topics, such as the methane emissions from livestock, the promise of AI technology, difficulties in gathering data, the use of methane in carbon credit programs, and current research and innovation. The review aims to improve knowledge and practices for climate change mitigation by highlighting the crucial role that accurate measurement and estimation methodologies play. It draws attention to the role that methane produced by livestock, particularly cattle, plays in climate change and stresses the need for precise measuring methods to be integrated into mitigation efforts.

**Abstract:**

This review examines the significant role of methane emissions in the livestock industry, with a focus on cattle and their substantial impact on climate change. It highlights the importance of accurate measurement and management techniques for methane, a potent greenhouse gas accounting for 14–16% of global emissions. The study evaluates both conventional and AI-driven methods for detecting methane emissions from livestock, particularly emphasizing cattle contributions, and the need for region-specific formulas. Sections cover livestock methane emissions, the potential of AI technology, data collection issues, methane’s significance in carbon credit schemes, and current research and innovation. The review emphasizes the critical role of accurate measurement and estimation methods for effective climate change mitigation and reducing methane emissions from livestock operations. Overall, it provides a comprehensive overview of methane emissions in the livestock industry by synthesizing existing research and literature, aiming to improve knowledge and methods for mitigating climate change. Livestock-generated methane, especially from cattle, is highlighted as a crucial factor in climate change, and the review underscores the importance of integrating precise measurement and estimation techniques for effective mitigation.

## 1. Introduction

Methane emissions are a significant contributor to greenhouse gas (GHG) emissions and play an important role in climate change mitigation efforts. Methane, as one of the primary GHGs, is a short-lived GHG that has a much higher global warming potential than carbon dioxide over a shorter time-period [1]. Methane is responsible for around 14–16% of total global GHG emissions, according to the Intergovernmental Panel on Climate Change [2,3]. As heat wave occurrences are predicted to occur more frequently and intensely over the planet, climate change is likely to make these problems worse [4]. The parties to the Paris Agreement agreed to limit global warming to 1.5 °C, requiring a reduction in agricultural methane emissions by 24–47% and the achievement of net-zero CO_2_ emissions by mid-century [5]. In addressing climate change, 195 countries have pledged to decrease greenhouse gas (GHG) emissions as part of the United Nations Framework Convention on Climate Change (UNFCCC) and have presented their national climate action strategies, known as their Nationally Determined Contributions (NDCs). The 195 parties affiliated with the Paris Agreement are obligated to report their national greenhouse gas inventories and progress towards achieving emissions reduction goals. Understanding and controlling methane emissions have become an essential priority as global concerns about climate change continue to rise. The cattle industry has emerged as a major contributor to methane emissions among many sources (e.g., pigs and chickens). Approximately 30% of methane emissions in the United States come from ruminants such as dairy and beef cattle (43.6 and 125.3 MMT, respectively). Therefore, ruminant emissions of GHGs have received significant attention in recent years [6]. Because of enteric fermentation and manure management procedures, livestock operations, especially beef and dairy production, are responsible for significant methane emissions [7,8,9].

**Figure 1:**
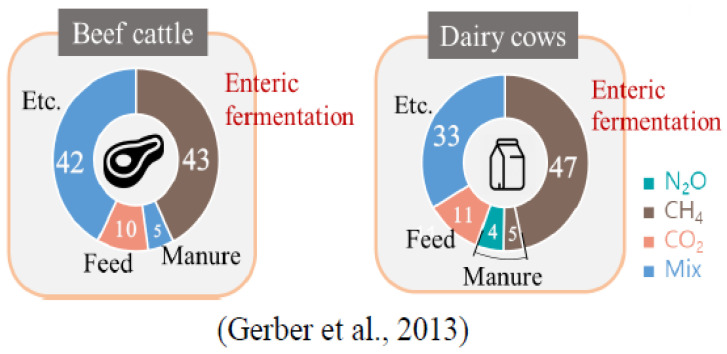
Enteric fermentation by beef and dairy cattle [11].

In recent years, there has been a growing realization of the relevance of methane emissions and the necessity for developing precise measurements in order to build effective climate change mitigation methods such as expanding the usage of renewable energy. It is noteworthy that not only enteric methane emissions but also natural processes, energy generation and usage, waste management, and agricultural activities all contribute to methane emissions. The livestock industry, particularly cow production, has been identified as a substantial contributor to global methane emissions among these sources [7,10]. Because of enteric fermentation, which occurs during the digestion process in the rumen of ruminant animals, livestock, notably beef and dairy cattle, are known to be major producers of methane [11] Figure 1.

**Figure 2:**
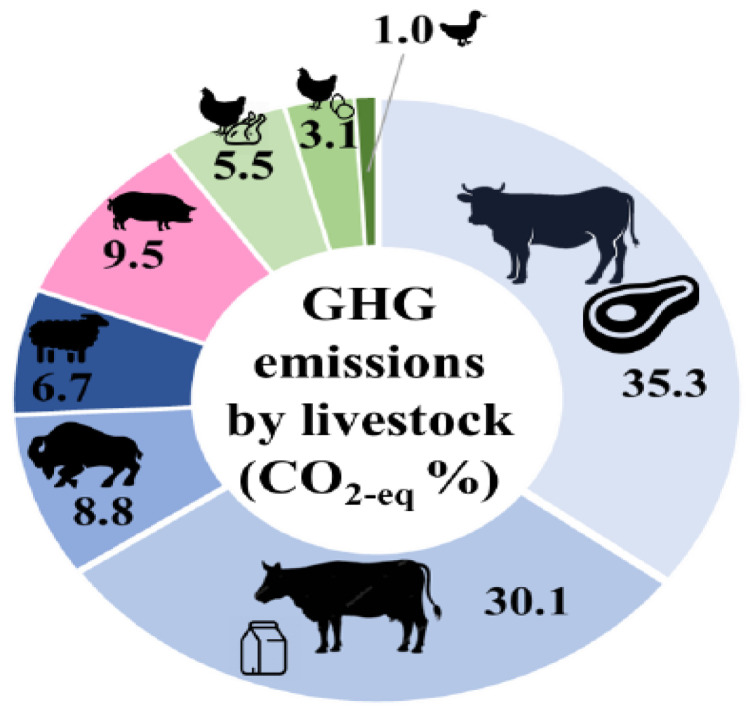
Contribution of GHG emissions from livestock industry.

The enteric fermentation process produces and emits methane gas into the atmosphere. According to research [11,12], the livestock industry emits around 7.1 gigatonnes of carbon dioxide equivalents of GHGs annually, or about 14.5% of all human GHG emissions worldwide. Small ruminants and buffalos produced 0.47 and 0.62 gigatonnes of carbon dioxide equivalents, respectively, compared to the 4.6 gigatonnes of carbon dioxide equivalents produced by cattle, of which 2.5 gigatonnes came from beef and 2.1 gigatonnes from dairy cattle. About 45% of the total carbon dioxide equivalent emissions from the two species of cattle was methane from enteric fermentation. Figure 2 illustrates the contribution of livestock to GHG emissions, with ruminants being the largest contributor. To enforce fair mitigation strategies, accurate estimation of methane emissions from the livestock industry is crucial. However, to implement such mitigation strategies, effective and reliable monitoring methodologies for quantifying methane emissions from cattle are necessary [2,9]. Various methods for measuring methane emissions from animals have been developed and used over the years. Direct measuring approaches such as respiration chambers, the Green-Feed system [13,14,15], sniffer technique, and open-circuit respiration systems [16] are among these methods, as are indirect measurement techniques such as the use of proxies and emission models [17,18]. Each approach has advantages and disadvantages, and the method chosen is determined by considerations such as accuracy, cost, and practicality in various circumstances.

Despite advances in measurement techniques, there is still need for improvement in methane emission estimation, notably in the cattle industry. Traditional estimation formulas, such as those employed in Japan, may fail to account for the distinct characteristics and conditions of various locations and cattle production systems. Ruminant production systems contribute different amounts of GHG emissions depending on the country and the region within that country [2]. Methane is a GHG that has been properly quantified, but this has led to concerns about how to report GHG inventories correctly and, perhaps more importantly, how to best control methane emissions. Already, the IPCC has provided methodologies to estimate GHG emissions [19]. The IPCC has suggested the conversion factor for converting gross energy intake into enteric CH_4_ energy (Ym) when calculating methane emission from ruminants, but IPCC data sets are not adequate to corroborate the data from various regions. For this reason, the IPCC encourages the development of country-specific methane estimation methods for enteric CH_4_ emissions. Hence, developing region-specific estimation methods is critical for increasing the accuracy of methane emission estimations [2,18].

Recent advances in artificial intelligence (AI) technology have demonstrated considerable promise in improving methane emission estimation. Multiple linear regression, support vector regression [20], random forest regression [21], and artificial neural networks (ANNs) [20] have all been used successfully to forecast methane emissions from cattle based on various input characteristics. Machine learning methods are used in these models to examine big datasets and find complex correlations between methane emissions and influencing factors [22].

Given the above information, the purpose of this review paper is to provide an overview of the importance of methane emissions in the context of climate change mitigation, the importance of accurate methane measurements, the role of the livestock industry in methane production, the challenges in estimating methane emissions, and the potential of AI technologies in improving methane emission models. Furthermore, the study will examine several data collection methods for methane emissions and investigate the on-farm application of monitoring methane emissions from cattle. This review study will add to a better knowledge of methane emissions and their implications for climate change mitigation methods in the livestock industry by integrating existing literature and research findings.

The paper is structured as follows: After the above introduction, Section 2 provides an overview of the CH_4_ emissions from the ruminant industry. It defines methane emission sources in livestock operations and the impact of livestock methane emissions on climate change. Section 3 discusses why it is necessary to develop accurate methods for estimating CH_4_ emissions from ruminants, with a focus on two approaches: animal-based and policy-based. Section 4 discusses approaches for methane emission estimation, such as the significance of precise methane emission estimates, methodologies to estimate methane emissions from ruminants, as well as the role of region-specific formulas. It summarizes the methodologies and challenges for collecting methane emission data and its estimation, highlights research gaps and future objectives in methane emission estimation, and emphasizes the significance of ongoing research and innovation to enhance our understanding of methane emissions and improve measurement techniques. The section also investigates the shortcomings of current estimation formulae, particularly in the context of the livestock industry, and underlines the importance of establishing region-specific formulas to increase the accuracy of methane emission estimations. Section 5 delves into more in-depth information on AI technology breakthroughs and their potential for enhancing methane emission prediction methods. This section investigates various AI-based models, such as multiple linear regression, support vector regression, random forest regression, and artificial neural networks (ANN), and highlights their applicability in predicting livestock methane emissions. The section covers the benefits of AI models in capturing complex correlations and patterns in huge datasets, resulting in more accurate estimates of methane emissions. Additionally, in this section, we also discuss the difficulties and issues involved in gathering data on methane emissions on farms. The part also discusses the significance of data quality and standardization in ensuring trustworthy and consistent results. Finally, Section 6 highlights the implications and future directions of methane emissions, the collection of data, and the use of advanced technology for the methane emission estimation and modeling.

Overall, this review paper aims to provide a comprehensive overview of methane emissions in the livestock industry, emphasizing the importance of accurate measurement and estimation methods. By synthesizing the existing literature and research findings, this paper seeks to contribute to the ongoing efforts to mitigate climate change by effectively managing methane emissions from livestock operations.

## 2. Methane Production in the Livestock Industry

### Methane Emission Sources in Livestock Operations

Livestock operations encompass a range of activities that contribute to methane emissions. Methane is produced and released during enteric fermentation, the process by which ruminant animals such as cattle and sheep digest their food [9,10,23,24]. Microorganisms in these animals’ rumens break down complex carbohydrates by anaerobic digestion, producing methane as a byproduct [8]. Furthermore, livestock manure management, including storage, processing, and disposal, can result in considerable methane emissions [1,19]. Anaerobic conditions in manure storage systems boost methanogenic bacteria activity, resulting in methane production [2,25].

The importance of cattle methane emissions in climate change cannot be overlooked. Enteric fermentation from ruminants accounts for about 90% of global methane emissions from livestock [10]. Given this, considering the differences in livestock categories, ruminants account for the largest share of livestock methane emissions in most countries [7]. Methane is a potent GHG, with a global warming potential that is more than 20 times that of carbon dioxide over a 100-year period [4]. Long-term livestock methane emissions have significant climate change implications, worsening the greenhouse effect and further disrupting the Earth’s climate system [11]. Methane emissions have a particularly strong warming effect in the short term, making it critical to address these emissions in order to prevent the near-term effects of climate change [10,11].

## 3. Need for Accurate Methane Estimation Methods

### 3.1. Need for Accurate Methane Emission Estimation (Animal Background)

Methane emissions from livestock farms must be accurately estimated in order to mitigate GHG emissions and to explore alternatives for reducing them. Precise quantification of methane emissions offers a baseline for measuring the efficacy of emission reduction efforts and enables the formulation of realistic targets [26,27,28]. Current methods for measuring CH_4_ emissions from ruminants show high accuracy compared to the respiration chamber method, which is considered the golden standard technique in quantifying the CH_4_ emissions from animals. However, respiration chambers also have the potential to over- or underestimate CH_4_ emissions due to their limitations, such as abnormal animal behaviors and decreased feed consumption [29]. The hand-laser methane detector (LMD) also has the potential to detect CH_4_ emissions from ruminants, accounting for atmospheric variations such as air humidity, wind speed, and atmospheric pressure [2]. Uncertainty is inherent in all measurement techniques due to random components such as changes in animal diets, management practices, and environmental conditions [30]. Current methods still have potential to over- or underestimate the baseline of CH_4_ emissions in ruminants due to their random factors. As a result, creating strong and accurate estimating methods is critical for addressing the climatic impact of livestock methane emissions.

### 3.2. Need for Accurate Methane Emission Estimation (Policy Background)

The Kyoto Protocol has established mandatory goals for reducing GHG emissions for nations that have endorsed the agreement. To accomplish the goal of reducing GHG emissions, countries must monitor and maintain records of their emissions and carbon market transactions. All Annex 1 countries are obligated to provide yearly national inventories of GHG emissions and removals, encompassing methane emissions from livestock (IPCC, 2006) [31]. In certain Annex 1 nations, producers in the livestock sector who decrease methane emissions have the opportunity to trade these reductions as carbon offsets within an emissions trading system.

Some policy approaches, such as incentive-based approaches, aim to address the necessity of reducing greenhouse gas concentrations in different countries by imposing transaction costs on producers, rendering participation unprofitable for small-scale operations [32,33]. Credits are quantified by calculating the carbon dioxide-equivalent (CO_2_e), and methane is recognized to have a greater global warming effect compared to CO_2_. Therefore, when calculating CO_2_e, methane is assigned a higher value to reflect its more potent impact on climate change. Consequently, methane emissions are important in the evaluation and allocation of carbon credits in emission trading schemes [32,33]. The basis for computing carbon credits can be formed by correctly monitoring and quantifying methane emissions.

Accurate methane measurements are critical for verifying and establishing the authenticity of carbon credits provided by methane reduction schemes. Methane emissions must be measured using trustworthy and established procedures to ensure consistency and comparability of data across projects and regions for their feasibility [17].

These measurements are used to verify the actual emission reductions achieved by the projects and to determine the quantity of carbon credits that can be issued. The most crucial aspect regarding the need for precise measurement of GHGs in the context of the carbon credit system is that inaccurate figures may lead to unreliable data. A past study reported that the current method for estimating methane emissions from offshore oil and gas production in the United Kingdom systematically and severely underestimates emissions [34]. Therefore, using accurate methods for methane measurement is not only essential for precise estimation but also crucial for the effective implementation of systems like carbon credits. Also, to improve the accuracy of estimation, verification processes, including independent third-party audits, are required to safeguard the carbon market’s integrity and openness [32].

In conclusion, methane measurements are critical in assessing and verifying carbon credits in emission trading schemes. Precise measuring methods for methane emissions are utilized to calculate carbon credits and are required to prove the legitimacy of emission reduction efforts. The potential for mitigating climate change through methane reduction initiatives can be realized by tying methane emission estimation to carbon credit evaluation.

## 4. Estimation Methods for Methane Emissions from Ruminants

### 4.1. Traditional Approaches for Methane Emission Estimation

Methane emission estimation in the cattle industry has traditionally depended on a variety of techniques, including direct measurement techniques and modeling approaches. On-site measurements employing chambers or portable analyzers to capture and quantify methane emissions from animals are used in direct measurement techniques [2,19,35]. These approaches produce reliable data, but they are sometimes time-consuming, labor-intensive, and logistically difficult, particularly for large-scale livestock operations [36]. On the other hand, modeling approaches estimate methane emissions using mathematical models that combine parameters such as animal population, nutrition, and management practices [37]. These models provide a more practical and cost-effective option, although they may have limits due to modeling simplifications and assumptions.

### 4.2. Direct Measurement Techniques

The main source of GHG emissions from ruminant livestock production systems is the methane created during enteric fermentation and manure processing and storage. However, there is no single, better method for quantifying methane in animal or dung waste [2]. Some approaches are more suited for small-scale situations, mostly because they were created with that goal in mind, whilst others were created from the start with large-scale applications in mind. However, this does not imply that all approaches are equivalent or that their quantification can be scaled up or down without introducing additional uncertainties or relying on underlying premises that might not apply in all circumstances [2]. Therefore, whether considering climate neutrality or global warming, it is difficult to give a firm evaluation of a production system’s sustainability (or resiliency for the purpose of inclusivity). Hill et al. [38] introduced different techniques to measure enteric methane, including respiration and accumulation chambers, hood and/or headbox systems, tracers, gas sensor capsules, in vitro techniques, open-path laser, unmanned aerial/ground vehicles (UAV/UGV, drones), satellite, computer models, and Light Detection and Ranging (LiDAR), and discussed their cost, applications, advantages and disadvantages in their article [2]. Storm et al. [36] and Zhao et al. [17] provided an in-depth review of the measurement techniques used for methane collection. Direct measurement techniques typically involve three different detection approaches, and information on these approaches is provided below: flux chambers, tunable diode laser absorption spectroscopy, and open-path laser spectroscopy. For instance, in the context of measuring methane emissions from cattle on-farm, open-path analyzers and closed-path analyzers offer real-time monitoring of methane concentrations in the cattle area [17,39]. Closed-path analyzers collect air samples and assess them in a controlled setting (Using flux chamber approaches), while open-path analyzers utilize infrared technology to measure methane concentrations along a defined path length [1,17].

Flux chamber: This method entails enclosing the source of the emission (e.g., livestock manure, wetlands) within a chamber and measuring the change in methane concentration over time. Based on the concentration change and chamber properties, the emission rate is computed.

Tunable diode laser absorption spectroscopy (TDLAS): TDLAS measures methane concentrations in the air by using laser beams at specific wavelengths. This non-invasive technology can be used to monitor methane emissions from industrial sites, landfills, and natural gas infrastructure in real time.

Open-path laser spectroscopy: Open-path spectroscopy uses laser beams that traverse the open air to measure methane concentrations. It provides spatially integrated measurements and is commonly used for monitoring methane emissions from large areas, such as agricultural fields and oil and gas facilities.

#### 4.2.1. Respiration Chambers

The respiration chamber method is a tried-and-true method for precisely measuring methane emissions from fermentation in the rumen and hindgut of animals [40]. Respiration chambers are considered the gold standard when measuring methane emissions from ruminants [41]. To compute methane emissions, the animal is enclosed in a sealed space while the airflow and concentration differences between the entrance and outlet air are measured. For accurate and repeated readings, automated sampling and gas analyzers are used. The technique has been used historically to examine gaseous exchange and the balance of energy in animal metabolism [2]. The advantages of respiration chambers are numerous. They enable the real-time tracking of methane flux, giving information on nocturnal emission patterns and prompt feedback on feed additives or nutrition [42,43]. The technique makes it possible to explore the connections between methane generation and a variety of elements, including animal traits, feed consumption, nutritional content, and feeding practices [44]. It enables energy partitioning, microbiological analysis, and precise measurements of methane emissions from ruminal and hindgut fermentation, which advances knowledge of methane generation mechanisms. There are limitations to take into account. The equipment needed for the technically challenging respiration chamber approach is expensive to build and maintain. The number of animals that can be studied at once is constrained by the chamber size. The training and acclimatization of the animals to the chambers requires labor and effort [42,43]. Measurements of methane made under controlled circumstances might not accurately represent animal emissions under various environmental situations. In addition, animals may consume less food in chambers than they would in grazing conditions.

#### 4.2.2. Sulfur Hexafluoride (SF_6_) Tracer Technique

Measuring the generation of methane in grazing and indoor ruminants is performed using the SF_6_ tracer approach [45,46]. An oral dose of the inert gas SF_6_ is administered into the rumen of the animal using a controlled-release permeation tube. Using a modified halter with a capillary tube attached to an evacuated collar worn around the animal’s neck, the methane and sulfur hexafluoride emissions are analyzed. The canister’s contents are examined using gas chromatography to determine the concentrations of methane and SF_6_ [47]. The ratio of the methane and SF_6_ concentrations inside the canister and the rate at which SF_6_ is released from the permeation tube are used to compute the methane emission rate [17,46]. The background methane and SF_6_ concentrations in the area around the animals are taken into consideration via a modified equation [17,46]. To increase the accuracy of the method, additional equipment is utilized to monitor ambient concentrations of methane and SF_6_ [45]. The SF_6_ method is useful for evaluating methane emissions from a large number of grazing animals at once since it does not need animal confinement, is affordable, and is non-invasive [47]. There are limitations to take into account, though. Since methane production can vary throughout the day but SF_6_ diffuses at a consistent rate, the notion that methane and SF_6_ fluxes through an animal’s nose and mouth are the same has been called into question [45]. The SF_6_ method can overestimate methane emissions when permeation tubes are deployed for an extended period of time [45]. After each study, it is critical to quantify the SF_6_ release rate and perform recovery tests for permeation tubes [48]. The accuracy of the findings may potentially be impacted by uneven accumulation of methane and SF_6_ concentrations in animal sheds and inadequate mixing of gases under stable nighttime conditions [48,49]. To make sure that animal welfare and behavior are not jeopardized, consideration should be given to the size, weight, and wearability of the SF_6_ equipment [48,49]. To determine the total methane output from the digestive tract, adjustments must be performed to account for rectum methane emissions [48]. In conclusion, the SF_6_ tracer methodology provides a useful way to calculate methane emissions from ruminants in both grazing and indoor situations. While there are benefits, such as non-invasiveness and the capacity to research numerous animals at once, there are also drawbacks to take into account, such as assumptions, accuracy under certain circumstances, and changes required for thorough methane estimation. The reliability and usefulness of the sulfur hexafluoride approach in methane emission studies can be improved by addressing these limitations and conducting additional research.

#### 4.2.3. GreenFeed

The automated head-chamber system known as GreenFeed was created by C-Lock Inc. to spot sample methane emissions and gaseous exchange in ruminants. It comprises a head-chamber system coupled with a mobile feeding station. The GreenFeed system has been compared to several techniques, including respiratory chambers [50,51], which have historically been thought of as the gold standard [51]. The GreenFeed system is an automated head chamber that continually calculates the animal’s gas emissions and analyzes gas concentrations when a visiting animal is detected by a proximity sensor inside the head chamber [52]. The system incorporates a number of data inputs, including ventilation, bait feed ingestion, gas concentrations, and automated animal identification using a radiofrequency identification ear tag [51,53]. The automated gas sampling procedure involves drawing air from the animal’s mouth and nose into a duct that measures airflow. Using a non-dispersive infrared sensor, a subsample is pulled into a gas analysis device to ascertain the methane content [52]. The measurements are normally performed three to seven times each day for each animal. The average daily methane emissions for each individual animal are then determined using the information gathered over a few days. The GreenFeed program regulates the timing and quantity of feed availability for each animal and makes sure that the measurements are distributed equally during a 24-h feeding cycle [48]. For the purpose of estimating methane emissions, the collected data are transferred to a cloud-based analytic system created by the GreenFeed manufacturer.

The GreenFeed system has the advantage of offering a portable and automated method for calculating each animal’s methane flux both indoors and when grazing [15,54]. In dairy cows and beef heifers, it has been proven to distinguish greater emitters from lower ones. Additionally, GreenFeed has demonstrated a strong association with methane production as determined by other techniques (e.g., respiration chambers and sulfur hexafluoride tracer gas). Nevertheless, there are several drawbacks to the GreenFeed system. Compared to other approaches like the respiration chamber and SF_6_, it shows considerable between-day and between-animal variability, which may limit its capacity to identify the effects of nutrition and animal factors on methane emissions. The need for a bait meal supplement can lead to differences between animals and interactions with dietary interventions, which can introduce further measurement variances. The voluntary nature of animal assessment and the impact of shifting wind direction and speed can have an impact on measurements in grazing experiments. Additionally, in a 24-h feeding cycle, methane emissions show a diurnal rhythm and are closely correlated with feed intake. In order to guarantee sufficient data throughout the feeding cycle for accurate assessment of daily methane emissions, it is important to regulate the quantity and timing of GreenFeed visits per animal.

#### 4.2.4. Sniffer Technique

Using this method, cows’ eructation (belches) is continually sampled for gases into a polyethylene sampling tube that is inserted in the feed trough of an automatic milking system [27,28]. An infrared methane concentration analyzer is then used to examine the gases that were sampled. The sniffer technique seeks to establish a correlation between daily methane production and methane concentration in eructation, as well as the corresponding frequency of eructation [17]. The sniffer technique has the benefit of being able to quickly and frequently assess methane concentrations from a large number of individual lactating dairy cows during normal milking under commercial settings. The sniffer technique has been found to provide a linear correlation between methane emission rate and methane production measured by the respiration chamber method, and the sniffer technique’s estimation of daily methane emissions is in good agreement with predictions based on milk yield and dairy cow body weight [27,28]. The sniffer technique can also distinguish between cows with high and low methane emissions. The sniffer technique, however, also has significant drawbacks. The precision of sniffers is significantly less [26] than that of respiration chambers [39] due to high within- and between-animal variation in the methane concentration in an animal’s breath. Comparing it to the respiration chambers and SF_6_ approaches, it shows more variation within and between cows. It is sometimes said to be less precise than the GreenFeed system and respiration chambers in predicting methane generation [15,54]. Dairy cow head movements in the feed trough, different feed trough designs, and sampling point positions can all have an impact on the accuracy of the sniffer technique by causing different air-mixing conditions and dilution effects of ambient air on the gas concentration in eructation. The association between the rate of methane production and the concentration of methane as determined by the sniffer technique may be disturbed by these factors, which introduce systematic inaccuracies. Additionally, the sniffer technique predicts methane emission values based on existing regression equations created using the RC approach rather than directly measuring methane flux or production [21]. As a result, various dietary circumstances may call for different formulae. Overall, the sniffer technique offers advantages in terms of its ability to measure methane concentrations in a large number of cows during routine milking and its correlation with methane production. However, it also has limitations related to variability, accuracy, and the need for regression equations based on the respiration chamber method. Further research and improvements are necessary to address these limitations and enhance the accuracy and applicability of the sniffer technique.

Other methods, including ventilated hood, facemask, and laser methane detector [16], are less used, and thus, we only mention their names here. For more information, the reviews by Storm et al. [36] and Zhao et al. [17] explain each method in detail. Studies also revealed that specific measurement technologies, data processing techniques, and uncertainty estimation strategies were frequently used in conjunction. This is due to the fact that some technologies and uncertainty assessment techniques are required for the application of data analysis methods, and some emission sources also call for particular instruments and data analysis techniques. The use of some precise measurement tools, on the other hand, did not always result in a clear shift in the level of uncertainty. All of these point to the possibility that methodologies and technologies might be thought of as a single system for estimating methane emissions and interact with one another to affect the final estimation [53].

Methane emissions are physically measured at the source or in the atmosphere using direct measurement techniques. These methods provide precise and real-time information on methane concentrations and emission rates.

### 4.3. Indirect Estimation Approaches

Indirect estimation methods infer methane emissions based on a variety of parameters and measurements connected to the source of the emissions. To estimate emissions, these approaches rely on mathematical models and statistical methodologies [17]. Some examples of common indirect estimate methods are as follows:

Methods based on inventory: To estimate methane emissions, these methods employ emission factors and activity data. Emission factors represent the average emissions per unit of activity (e.g., livestock population, fuel consumption), whereas activity data provide the frequency and volume of activities that contribute to emissions. This method is widely used to estimate emissions from large-scale sources such as livestock operations and trash disposal.

Remote sensing: Remote sensing techniques determine methane concentrations and emissions across vast areas by using satellite or aircraft data. These data, in conjunction with atmospheric modeling and statistical methodologies, can be used to estimate emissions from a variety of sources, including natural wetlands, landfills, and oil and gas infrastructure.

#### 4.3.1. Model Approach to Estimation of Methane Emissions from Ruminants

In Table 1, we provided a list of models of methane emission from ruminants (beef cattle, dairy, Holstein cattle). Estimation of enteric methane emissions from ruminants is important for assessing dietary mitigation strategies [9,23,24]. Measuring methane emissions from individual animals requires expensive equipment and advanced technologies. Normally, models can explain the relationship between nutrient intake and methane production by adopting mathematical methods to describe rumen fermentation biochemistry [36]. Presently, many countries accept the IPCC (2006) Tier 1 or Tier 2 methodologies to report their national inventories of GHG emissions. The Tier 2 model only considers the gross energy intake (GEI) and energy to methane conversion ratio (Y_m_) to estimate the methane emissions from ruminants, which can lead to inaccurate measures in predicting methane emissions when diets have different nutrient composition. Hence, in improving the accuracy of methane emissions models, it is necessary to consider region-specific methane emission factors [7]. Currently, region-specific methane estimation equations are being developed in some geographical areas [10].

To increase the accuracy of methane equations, various emission factors (e.g., age, breed, country-specific factors) are calculated for the methane estimations [55]. Models developed to date are normally based on feed intake [23,56,57]. These models do not adequately account for the effect of other dietary factors. Therefore, recently developed models account for dietary factors such as lipid supplementation, neutral detergent fiber (NDF), organic matter digestibility (dOM), and starch [8,12,58]. They consider various dietary factors to compensate for the model accuracy and to improve the prediction of methane emissions under different mitigation strategies that have been proposed. Also, mechanistic and regression model approaches (linear and non-linear) are being considered to develop equations of methane emissions from ruminants [56,59]. Variables for explaining the models for methane emissions were sufficiently comprehensive, and multiple-regression analysis was adopted to predict the methane emissions from ruminants [57,60]. All methods have different purposes, and none of them are applicable in all aspects. To develop more accurate models, it is necessary to identify the variables that can improve precision and incorporate various approaches.

#### 4.3.2. Incorporating Regional Variables for Developing Accurate Methane Estimation Models

The IPCC (2006) suggested a tier (Tier 1, Tier 2, and Tier 3) approach for national GHG inventories in the context of livestock, with successive tiers providing increased accuracy with the complexity of the approaches. The IPCC recommended developing region-specific emission factors to calculate CH_4_ emissions from ruminants.

The Tier 1 method involves default values for emission factors, and the data are based on previous studies, presenting the emission factors by region to facilitate ease of use. When opting for the Tier 1 method, readily available data on animal populations are needed, and recommendable to the countries where enteric fermentation is not a main source of GHG emissions. The IPCC (2006) [31] guidelines provide default emission factors for ruminants, organized into eight different regions to account for variations in cattle characteristics among regions.

The Tier 2 method is an improved method that requires information about animal categories, feeding and production systems, and manure management. The Tier 2 method is currently used for inventories in most industrialized countries. When calculating the CH_4_ from enteric fermentation by adopting the IPCC Tier 2 method, calculation is based on the gross energy intake (GEI) and conversion rate of gross energy intake into methane energy (Y_m_), which can be chosen according to the level of productivity and diet characteristics. The IPCC data derived from Niu et al. [61] mainly incorporates European research institutes and does not encompass other regions or countries. Diet characteristics in past reports can have differences in other countries (Mediterranean region) [62,63]; hence, the IPCC encourages the development of country-specific Y_m_ factors to enhance the accuracy of enteric CH_4_ estimation.

Tier 2 Emission factor = GE × (Y_m_/100 × 365 days/year)/55.65 MJ/kg CH_4_ (1)

The Tier 3 method is applicable to a national inventory when comprehensive knowledge of all factors influencing enteric and manure CH_4_ emissions is available and can be considered. A thorough and precise depiction of feeding systems is essential, encompassing crucial details such as the energy conversion factors utilized for estimating the energy requirements of the animals.

In the case of measuring methane within a regional scope, the measurement values may vary based on the climatic conditions prevailing during the measurement period in that particular region [2]. To develop an accurate formula for estimating methane emissions by incorporating regional variables, we integrate relevant factors to create a precise and specific formula. These variables should reflect differences in statistical, physiological, and behavioral characteristics identified in specific-country cattle populations. A past study reported that specific variables (Korea) relating to breed traits, feeding methods, specific environments, and management systems can be determined by collecting data on methane emissions and conducting bio-specimen analysis on beef and dairy cattle [55]. The inclusion of these Korean factors in the estimation formula aids in the reduction of errors and the accuracy of methane emission projections in the local context. Collaboration between researchers, policymakers, and industry stakeholders is critical for fully realizing the potential of AI technology in methane emission estimation. Innovative ways to overcome problems and ensure the successful on-farm detection of methane emissions from cattle can be developed by promoting collaboration and knowledge exchange. This collaboration can also help to standardize protocols and methodologies, allowing for data comparability and synthesis across research and locations.

Finally, incorporating AI technology into methane emission estimation holds potential for furthering our understanding of emissions in the livestock industry. We can increase our ability to estimate and mitigate methane emissions by developing reliable models, improving data collection and processing procedures, and implementing monitoring technology on farms. We may contribute to sustainable and environmentally responsible livestock production practices while limiting the influence of methane on climate change by tackling the hurdles and utilizing the benefits of AI-based approaches.

### 4.4. Challenges and Considerations for Methane Estimation in Ruminants

Traditional approaches have been useful in calculating methane emissions, but they have limits. Direct measuring approaches can be time-consuming and offer data that are not always representative of the total animal population [64]. Furthermore, the use of modeling methodologies necessitates appropriate input parameters, which can be difficult to obtain, thus contributing to uncertainty in estimations [65]. Methane estimation techniques may have difficulty capturing the dynamic character of methane emissions, which can fluctuate based on factors such as feed composition, animal behavior, and environmental circumstances [66].

Collecting accurate and trustworthy methane emission data presents numerous issues and must be approached with caution:

Spatial and temporal variability: Methane emissions can fluctuate both spatially and temporally, making it difficult to capture the entire spectrum of emissions. Important considerations include developing sampling procedures that effectively capture variability and accounting for seasonal and diurnal swings.

Measurement precision and sensitivity: Methane concentrations in the atmosphere are frequently low, necessitating precise monitoring techniques to reliably identify and quantify emissions. It is critical to ensure the precision and sensitivity of instruments in order to collect trustworthy data.

Measurement infrastructure: Deploying measurement tools and establishing monitoring networks can be difficult logistically, especially in remote or inaccessible places. Regular maintenance and calibration of the instruments are required for accurate and consistent measurements.

Source attribution: It can be difficult to identify and quantify methane emissions from individual sources in complicated ecosystems. Combining numerous measurement techniques, modeling methodologies, and complementing data sources can increase the accuracy of source attribution.

Standardization and quality assurance: It is critical to develop standardized protocols, techniques, and quality assurance procedures to ensure consistency and comparability of methane emission data across studies and locales.

Addressing these challenges and taking into consideration these factors in methane data collection efforts are crucial for generating reliable and robust emission estimates, supporting effective mitigation strategies, and accurately measuring the climatic impact of methane emissions.

Zhao et al. [17], in their review of the application of various methane estimation measurement methods, concluded that the correct and successful use of methane emission measurement methods depends on the objectives of the studies and the mechanism of each method. Respiration chambers and head enclosures are accurate enough to determine emission factors for IPPC inventory reporting, but not for use with grazing animals. The sulfur hexafluoride tracer technique can be used in grazing situations, but feed intake relies on indirect prediction. The short-term techniques (i.e., GreenFeed, Sniffer, Facemask, LMD and PAC) offer potential opportunities to identify high and low methane emitters in a large group of animals for breeding purposes, and thus, research is needed to improve their reliability and repeatability [55]. Overall, ideal methane measurement techniques should be accurate, rapid, cost-effective and automated, with an understanding of animal behavior and welfare that allows measurement of animals in their practical production environment.

**Table 1:** List of models of methane emission from ruminants (Beef cattle, Dairy, Holstein cattle).

Year	Equation ^1^	r^2^	Reference
2003	Methane (MJ/d)		[67]
	(1): = 5.93 (SE 1.60) + 0.92 (SE 0.08) × DMI (kg/d)	0.60	
	(2): = 8.25 (SE 1.63) + 0.07 (SE 0.007) MEI (MJ/d)	0.55	
	(3): = 7.30 (SE 1.58) + 13.13 (SE 3.41) N (kg/d) + 2.04 (SE 0.41) ADF (kg/d) + 0.33 (SE 0.18) Starch (kg/d)	0.57	
	(4): = 1.06 (SE 2.41) + 10.27 (SE 3.59) Dietary forage proportion + 0.87 (SE 0.074) DMI	0.61	
2007	Beef cattle		[56]
	(1): CH_4_ (MJ/d) = 2.94 (±1.16) + 0.0585 (±0.0201) × ME intake (MJ/d) + 1.44 (±0.331) × ADF (kg/d) − 4.16 (±1.93) × lignin (kg/d)	0.85	
	(2): CH_4_ (MJ/d) = 0.183 (±1.85) + 0.0433 (±0.0170) × ME intake (MJ/d) + 0.647 (±0.244) × NDF (kg/d) + 0.0372 (±0.0186) × forage (%)	0.74	
	Dairy		
	(1): CH_4_ (MJ/d) = 2.16 (±1.62) + 0.493 (±0.192) × DMI (kg/d) − 1.36 (±0.631) × ADF (kg/d) + 1.97 (±0.561) × NDF (kg/d)	0.63	
	(2): CH_4_ (MJ/d) = 1.64 (±1.56) + 0.396 (±0.0170) × ME intake (MJ/d) + 1.45 (±0.521) × NDF (kg/d)	0.59	
	Combined		
	(1): CH_4_ (MJ/d) = 3.69 (±0.993) + 0.543 (±0.132) × DMI (kg/d) + 0.698 (±0.247) × NDF (kg/d) − 3.26 (±1.56) × lignin (kg/d)	0.71	
	(2): CH_4_ (MJ/d) = 3.41 (±0.973) + 0.520 (±0.120) × DMI (kg/d) − 0.996 (±0.447) × ADF (kg/d) + 1.15 (±0.321) × NDF (kg/d)	0.67	
2013	CH_4_ production (MJ/d)		[68]
	(1): = 1.36 (±0.10) × DMI − 0.125 (±0.039) × FA − 0.02 (±0.012) × CP + 0.017 (±0.005) × NDF	0.77	
	(2): = 1.23 (±0.08) × DMI − 0.145 (±0.039) × FA + 0.012 (±0.005) × NDF	0.75	
	(3): = 1.39 (±0.06) × DMI − 0.091 (±0.036) × FA	0.70	
	(4): = 1.26 (±0.03) × DMI	0.66	
	(5): = 738 (±54) × DMI_BW − 0.145 (±0.044) × FA + 0.013 (±0.005) × NDF	0.68	
	(6): = 0.0026 (±0.0004) × rdNDF + 0.0020 (±0.0004) × rdstarch + 0.0032 (±0.0004) × rdrestCHO	0.59	
2013	(1): CH_4_-E/GE (kJ/MJ) = −0.6 (±12.76) − 0.70 (±0.072) × DMIBW (g/kg) + 0.076 (±0.0118) × OMDm (g/kg) − 0.13 (±0.020) × EE (g/kg of DM) + 0.046 (±0.0097) × NDF (g/kg of DM) + 0.044 (±0.0094) × NFC (g/kg of DM)	RMSE (3.26 kJ/MJ)	[68]
	(2): CH_4_ (L/d) = −64.0 (±35.0) + 26.0 (±1.02) × DM intake (kg/d) − 0.61 (±0.132) × DMI^2^ (centered) + 0.25 (±0.051) × OMDm (g/kg) − 66.4 (±8.22) × EE intake (kg of DM/d) − 45.0 (±23.50) × NFC/(NDF + NFC)	RMSE (21.1 L/d)	
	(3): CH_4_-E/GE = 0.96 (±0.103) × predicted CH_4_-E/GE + 2.3 (±7.05)	RMSE (3.38 kJ/MJ)	
2014	Holstein cattle		[69]
	6 months old		
	(1): CH_4_ (g/day^−1^) = 0.341_(0.128)_ BW (kg) + 30.7_(22.7)_	0.26	
	(2): CH_4_ (g/day^−1^) = 26.0_(4.22)_ DM intake (kg day^−1^) − 11.1_(17.2)_	0.67	
	(3): CH_4_-E (MJ day^−1^) = 0.765_(0.0112)_ GE intake (MJ day^−1^) − 0.660_(0.868)_	0.72	
	12 months old		
	(1): CH_4_ (g day^−1^) = 0.319_(0.0983)_ BW (kg) + 57.0_(31.6)_	0.34	
	(2): CH_4_ (g day^−1^) = 16.7_(2.14)_ DM intake (kg day^−1^) + 47.2_(14.4)_	0.76	
	(3): CH_4_-E (MJ day^−1^) = 0.048_(0.0054)_ GE intake (MJ day^−1^) + 2.53_(0.721)_	0.80	
	18 months old		
	(1): 0.234_(0.122)_ BW (kg) + 59.5_(60.3)_	0.12	
	(2): 14.1_(4.68)_ DM intake (kg day^−1^) + 73.3_(34.0)_	0.30	
	(3): 0.032_(0.0121)_ GE intake (MJ day^−1^) + 4.89_(1.84)_	0.24	
	22 months old		
	(1): 0.275_(0.0675)_ BW (kg) + 32.0_(38.4)_	0.45	
	(2): 13.3_(4.28)_ DM intake (kg day^−1^) + 79.4_(35.2)_	0.31	
	(3): 0.032_(0.0127)_ GE intake (MJ day^−1^) + 5.15_(2.10)_	0.22	
2016	Enteric methane emissions (EME; MJ/day)		[58]
	(1): = 0.242 (×0.073) + 0.0511 (×0.0073) × digestible energy intake	0.83	
	(2): = −1.04 (±0.271) + 2.21 (±0.395) × neutral detergent fiber intake × 2.42 (±1.10) × ether extract (EE) intake + 1.456 (±0.323) × non-fiber carbohydrate intake + 0.0208 (±0.0039) × OM digestibility at maintenance level of feeding (OMDm) − 0.513 (±0.137) × feeding level (FL)	0.82	
	(3): = −0.885 (±0.154) + 0.809 (±0.0867) × dry matter intake − 0.397 (±0.0494) × FL + 0.0198 (±0.0022) × OMDm + 2.04 (±0.234) × acid detergent fiber intake −8.54 (±0.548) × EE intake	0.88	
	(4): = 1.721 (±0.151) × {1 − exp(−0.0721 (±0.0092) × metabolizable energy intake)}	0.79	
2016	Single linear prediction of methane emissions from nonpregnant nonlactating dairy cows		[70]
	CH_4_ (methane emissions) (kg/d)		
	(1): = 50.67_(14.03)_ + 19.95_(2.16)_ DMI (kg/d)	0.67	
	(2): = 50.85_(13.52)_ + 21.63_(2.28)_ OMI (kg/d)	0.68	
	(3): = 73.15_(16.01)_ + 20.56_(3.10)_ DDMI (kg/d)	0.61	
	(4): = 63.19_(15.31)_ + 23.78_(3.15)_ DOMI (kg/d)	0.62	
	CH_4_-E (methane energy output) (MJ/d)		
	(1): = 2.727_(0.807)_ + 0.061_(0.007)_ GEI (kg/d)	0.68	
	(2): = 4.341_(0.887)_ + 0.060_(0.009)_ DEI (kg/d)	0.63	
	(3): = 6.110_(0.805)_ + 0.047_(0.010)_ MEI (kg/d)	0.62	
2020	CH_4_ emissions (g/day)		[65]
	(1): = 0.44 (±0.02) × BW	0.63	
	(2): = 213 (±21.0) + 6.26 (±0.85) × milk	0.57	
	(3): = 117 (±7.97) + 36.1 (±12.1) × ADG	0.14	
	(4): = 19.4 (±7.25) + 16.7 (±1.09) × DMI	0.78	
	(5): = 63.8 (±11.6) + 0.96 (±0.07) × GEI (for dairy cattle) 63.8 (±11.6) + 0.72 (±0.10) × GEI (for mature cattle)	0.79	
	(6): = 68.1 (±13.5) + 12.4 (±1.99) × DMI − 0.53 (±0.26) × EE	0.63	
	(7): = 111 (±18.6) + 23.0 (±2.35) × dDMI − 31.3 (±9.41) × FL − 0.08 (±0.04) × NFC	0.78	
	(8): = 17.0 (±0.99) × DMI + 0.03 (±0.01) × NDF	0.81	
	(9): = 18.1 (±1.23) × DMI + 0.33 (±0.15) × Forage − 0.30 (±0.20) × dOM	0.80	

^1^ Equation parameters are ±SE, FA = fatty acids, NDF = Neutral detergent fiber, ADF = Acid detergent fiber, CP = Crude protein, DMI = Dry matter intake (kg/day), ME intake (MJ/d) = Metabolizable energy intake, rd_ = rumen degraded nutrient (g/d), td_total digested amounts, rdrestCHO = rest fraction of carbohydrates calculated as the residue when subtracting protein, fat, starch, NDF and fermentation products from organic matter, OMDm = OM digestibility determined at a maintenance level of feeding = OMD + 1.83 × (DMIBW − 10), GE = Growth energy, DMIBW = Dry matter intake per kg body weight, dOM = digestibility of OM, DDMI = Digestible dry matter intake, DOMI = Digestible organic matter intake, DEI = Digestible energy intake, dNDF = digestibility of NDF, DDMI = digestible DM intake (kg/day), CH_4_-E = CH_4_ energy, BW = animal body weight (kg), FL = feeding level (%BW), Forage = forage percentage in the diet, GEI = gross energy intake (MJ/day), RMSE = Root mean square error.

## 5. One of the Methods for Increasing the Accuracy of Methane Emissions Models for Ruminants: AI Technology

### 5.1. The Role of AI Technologies in Advancing Methane Emission Estimation

The rise of artificial intelligence (AI) technology has created new opportunities for better estimating methane emissions in the cattle industry. Machine learning and deep learning algorithms [71], for example, have shown promise in dealing with complicated datasets and identifying hidden patterns in methane emissions data [22,72]. These technologies are capable of analyzing enormous amounts of data, such as animal-related metrics, feed composition, and environmental conditions, in order to construct more accurate and robust estimation models [1,22]. AI-based systems may also include real-time data streams, enabling continuous monitoring and updating of methane emission estimations [1].

### 5.2. Data Collection and Processing Techniques

Accurate estimation of methane emissions requires systematic data collection and processing techniques. This includes documenting animal characteristics (such as breed, age, and weight), meal composition, feeding procedures, and environmental conditions (such as temperature and humidity) [39,73,74]. Collecting the bio-specimen data can improve the accuracy of methane estimation models for ruminants. Ruminants’ rumen fluid, feces, or breath can be collected and evaluated for methane content [2]. Rumen fluid analysis, in particular, gives information about the rumen’s microbial population and fermentation activities, which are linked to methane production [75,76,77]. Stable carbon isotope analysis, for example, can be used to distinguish methane produced by microbial fermentation from methane produced by other sources [72,78]. A more comprehensive understanding of methane production and potential mitigation measures can be acquired by combining bio-specimen analysis with emission measurements. Electronic data capture systems or mobile applications can help with on-farm data collection, allowing for more efficient and uniform data collection [1,17]. Additionally, integration with on-site meteorological stations or weather data sources improves the contextual knowledge for estimating emissions.

Jeong et al. [1] provided three main data collection methods based on AI models and discussed their output in detail. The categories included (1) aerial imagery processing, (2) deep learning image segmentation, and (3) dairy facility area estimation.

Briefly, aerial imagery processing refers to the utilization of aerial imagery data for various applications and analysis. It involves the collection, processing, and interpretation of photographic images captured from aerial platforms. Aerial imagery processing techniques, such as photogrammetry, are used to extract reliable information about physical objects and the environment by recording, measuring, and interpreting photographic images. This process allows for the generation of two-dimensional (2D) or three-dimensional (3D) digital models of objects. In their paper [1], aerial imagery processing is specifically used for training an AI model to estimate methane emissions from dairy operations.

Deep learning image segmentation refers to the process of dividing an image into multiple segments or regions based on certain characteristics or properties of the pixels [72]. It is a technique used in computer vision and digital image processing to simplify and analyze images more effectively [2]. In the given context [1], deep learning image segmentation is specifically mentioned in relation to identifying dairy facilities at the pixel level on a National Agricultural Imagery Program (NAIP) image. The approach mentioned involves using deep learning (DL) methods, which are a subset of machine learning and rely on artificial neural networks (ANNs) to imitate the processing of the human brain [20]. Deep learning image segmentation enables the identification and classification of different objects or regions within an image at the pixel level. In this case, the objective is to predict the pixel-level location of dairy farms in the image, rather than just determining if the image contains a particular object. It allows for more detailed and precise analysis, such as calculating the dairy farm’s spatial area for emission estimation purposes [78]. The technique mentioned in the provided information utilizes a convolutional neural network (CNN) as a deep learning architecture for image segmentation [20]. Specifically, the researchers applied the U-Net architecture, which is known for performing well with a relatively small number of training datasets. U-Net was initially developed for medical imaging applications and was adapted for the abovementioned study. Overall, deep learning image segmentation plays a crucial role in various fields, including medical imaging, self-driving cars, satellite imaging, and other computer vision applications. It allows for a more granular analysis and understanding of images by assigning labels to individual pixels, enabling the extraction of complex and abstract features.

Dairy facility area estimation refers to the process of determining the spatial area occupied by dairy facilities, including free stall barns and open lots [26,27]. This estimation is used as a basis for estimating the dairy population within the facility, which is then utilized for emission estimation in combination with emission factors (EFs). In practice, a deep learning (DL) model is employed to identify individual dairy pixels within an image tile. It is worth noting that multiple image tiles can form a single facility, and by combining the boundaries from these tiles, individual facility boundaries are constructed to estimate the facility-level population [1,20]. To facilitate further analysis for dairy population and emission estimations, the spatial areas calculated from clusters of identified dairy pixels are aggregated into a grid with a resolution of 0.1° (approximately 10 km). This gridded area information is then utilized in subsequent analyses for dairy population and emission estimations. The study by Marklein et al. [79] utilized a spatial resolution of 0.1° for comparison with other spatial inventories, including CALGEM, although their native resolution was at the facility level. This comparison aims to assess the accuracy and reliability of the gridded product derived from the dairy facility area [79]. Therefore, dairy facility area estimation involves the use of deep learning models, high-resolution imagery, and spatial analysis techniques to identify dairy facilities, estimate their spatial areas, and scale the population at the facility level for emission estimation purposes.

### 5.3. Pre-Processing and Normalization of Methane Emission Data

Prior to analysis, methane emission data must be pre-processed and normalized. Data cleaning, outlier detection, and missing value management are all part of pre-processing procedures [17,23]. Data from multiple sources or scales can be compared using normalization approaches such as feature scaling or normalization to a reference value [23]. RobustScaler and Min-Max normalization methods can be used to alter the range and distribution of methane emission data, allowing for more effective analysis and modeling [17,23]. These steps help to improve the accuracy and dependability of methane emission estimation.

Overall, detecting methane emissions from cattle on-farm requires the use of portable measurement equipment, bio-specimen analysis, meticulous data collection and processing, and the addition of important region-specific variables. These steps help to produce precise and dependable estimations of livestock methane emissions. The use of portable methane emission measurement equipment enables on-site monitoring and real-time data collection, offering significant insights into individual animal emissions as well as herd emissions. These measurements are supplemented by bio-specimen analysis, which provides information on rumen microbial activity and methane generation mechanisms [80]. Proper data collection and processing techniques ensure that the collected data are of high quality and consistency, while pre-processing and normalization methods help to standardize the data for accurate analysis and modeling. Finally, using region-specific variables such as breed characteristics, environmental specifics, feeding habits, and management techniques, guarantees that the methane emission estimation formula is matched to the local context, reducing errors and enhancing prediction accuracy. Stakeholders in the cattle industry can acquire a better understanding of methane emissions and establish effective mitigation and sustainable management plans by applying these on-farm measurement and analysis tools.

Various machine learning techniques are used in the construction of models for estimating methane emissions. For example, multiple regression allows the development of a linear equation that represents the link between methane emissions and many input factors. The coefficients of the regression equation represent the contribution of each variable to methane emissions in this technique [81]. Support vector regression (SVR) and other supervised learning models can also be used to capture nonlinear correlations between factors and methane emissions [21,56]. These models are intended to learn patterns and correlations from training data and generate predictions based on previously unknown data.

Once the models are trained, they can be used to predict methane emissions by inputting the relevant variables. These predictions play a vital role in assessing and monitoring the environmental impact of livestock operations [82].

### 5.4. Model Performance Evaluation and Interpretation

To ensure the accuracy and trustworthiness of methane emission estimation models, their performance must be extensively assessed. To test the prediction performance of the models, various evaluation metrics such as mean squared error (MSE), root mean squared error (RMSE), and coefficient of determination (R-squared) are often used [59]. These measures provide useful information about the models’ capacity to capture variance in methane emissions as well as their overall accuracy.

Understanding the factors that drive methane emissions is also dependent on how the models are interpreted. The coefficients in multiple regression models provide information regarding the amount and direction of the relationships between factors and methane emissions [23]. Support vector regression models, on the other hand, use support vectors to identify the data points that have the most influence on the model’s decision limits [24,59]. Such interpretations aid in the identification of crucial variables and the understanding of the underlying mechanisms that drive methane emissions in livestock operations.

### 5.5. Artificial Neural Networks (ANN) for Complex Estimation

Artificial neural networks (ANNs) are powerful models that can recognize complicated correlations and nonlinear patterns in data [20]. ANNs are made up of interconnected nodes (neurons) that are structured into layers, which include an input layer, one or more hidden layers, and an output layer. Each node applies an activation function to its input and forwards the outcome to the next layer. Weights govern the strength of connections between nodes, which are modified during the training phase to maximize the model’s performance. The key distinction between the proposed ANN model and conventional inventory-based models is the ANN model’s requirement for a slightly smaller number of input parameters, which have a far wider availability. This makes it possible to use the ANN model to forecast methane emissions even in countries and regions without the full set of input parameters that are often required by conventional models based on activity levels and emission factors [20].

### 5.6. Model Creation and Architecture

To develop an ANN model for estimating methane emissions, the model’s design must be carefully considered. This entails establishing the number of layers and nodes within each layer, as well as choosing appropriate activation functions. The variables impacting methane emissions should be represented in the input layer, while the estimated methane emissions should be shown in the output layer. To enhance the model’s performance, the number of nodes in the hidden layers can be selected through experimentation and tuning [1].

### 5.7. Hyperparameter Tuning and Training

Optimizing parameters such as learning rate, batch size, and number of epochs to improve the model’s generalization and prediction performance is a critical stage in training ANNs [20]. Grid search and randomized search techniques can be used to investigate alternative combinations of hyperparameters and determine the ideal configuration [1]. Once the hyperparameters have been determined, the model can be trained using appropriate optimization techniques such as stochastic gradient descent (SGD) or the Adam optimizer. During the training process, the ANN’s weights are iteratively modified to minimize the difference between the predicted and actual methane emissions in the training dataset. This approach is repeated until the model converges or meets a predetermined stopping threshold [20].

### 5.8. Model Validation and Weight Analysis

Following training, the ANN model must be validated using distinct validation datasets to determine its generalization capabilities. On the validation dataset, performance metrics like MSE or RMSE can be derived to assess the model’s accuracy [20,21]. This stage ensures that the model can estimate methane emissions from unseen data and provides an indication of its trustworthiness in real-world circumstances. Another important part of evaluating ANN models is weight analysis. By evaluating the weights provided to the connections between nodes, valuable insights into the importance and contribution of various variables in predicting methane emissions can be obtained [7]. The analysis assists in identifying the major elements causing methane emissions in cattle operations and improves our understanding of the underlying processes.

**Figure 3:**
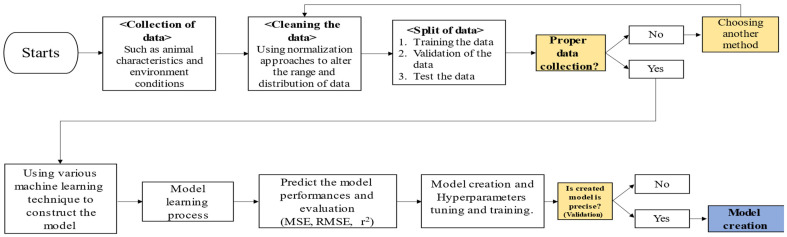
Flow chart of developing AI-technology-based on-farm application model for measuring methane emissions from cattle.

In conclusion, the development and evaluation of models for estimating methane emissions requires the use of a variety of approaches such as multiple regression, support vector regression, and ANNs [21]. These models provide greater precision and flexibility in capturing the complex linkages and nonlinear patterns involved with methane emissions. Proper model performance evaluation and interpretation ensures model dependability and aids in identifying key variables and mechanisms controlling methane emissions. We can increase our understanding of methane emissions in the cattle industry and contribute to effective mitigation methods by incorporating modern AI technology and investigating novel ways. Figure 3 provides a schematic of the process used to develop the methane emission equation.

### 5.9. Benefits and Challenges of AI-Based Approaches

The use of AI-based techniques for estimating methane emissions has various advantages. For starters, these techniques have the potential to improve estimation accuracy and precision by considering a greater range of factors and their interactions [82,83,84]. Second, AI approaches can improve the scalability and efficiency of estimation procedures, allowing for broader spatial and temporal scale assessments [85]. Furthermore, AI-based techniques may adapt to and learn from fresh data, resulting in ongoing refinement and development of estimation models [86].

However, there are several limitations related to using AI to estimate methane emissions. Data availability and quality are critical issues because AI models rely largely on large and consistent datasets for training and validation [87]. Integration of disparate data sources and data format harmonization are technological problems that necessitate appropriate data pretreatment and standardization [88]. The interpretability and transparency of AI models are also crucial concerns to assure the accuracy and acceptability of the estimated results [89,90].

Overall, the employment of AI technology in methane emission estimation shows a lot of promise in terms of enhancing accuracy, scalability, and efficiency [22]. However, more research and development are required to solve the obstacles and fully harness the benefits of AI-based systems in the context of estimating livestock methane emissions. One of the most important advantages of AI technologies is their capacity to manage big and complex information, which is especially important in the cattle industry, where a variety of factors influence methane emissions. AI-based techniques may efficiently capture nonlinear correlations and interactions among numerous variables by employing machine learning algorithms, resulting in more precise estimations [22,91]. Furthermore, AI technologies provide the benefit of constant learning and adaptation. AI models may be updated and adjusted as more data become available and new insights are discovered, allowing them to perform better over time. This adaptability is critical in the context of estimating methane emissions as our understanding of the underlying variables controlling emissions evolves. However, there are still difficulties in implementing AI-based techniques. Data quality and availability are significant considerations that must be addressed. For accurate model training, high-quality and representative datasets are required. Furthermore, incorporating AI technology into existing cattle operations may necessitate the removal of technical, logistical, and financial constraints.

## 6. Implications and Future Directions

### 6.1. Advancing On-Farm Methane Emission Monitoring Technologies

On-farm methane emission monitoring technology advancements have substantial consequences for the cattle industry and environmental management [57]. The introduction of portable methane emission measurement technology has made on-site data collection more accurate and efficient. Further research and development in this field can concentrate on improving the portability, usability, and cost of monitoring technology. This would allow farmers to employ the technology more widely and promote frequent and routine detection of methane emissions from livestock activities.

### 6.2. Integration of AI and IoT for Real-Time Methane Emission Monitoring

The combination of artificial intelligence (AI) and the Internet of Things (IoT) opens up new possibilities for monitoring methane emissions on farms in real time. AI algorithms can analyze continuous data streams from methane sensors and offer farmers rapid feedback, allowing for prompt action and mitigation. Remote monitoring of methane emissions across several locations can be facilitated by IoT-based sensor networks, offering a comprehensive understanding of emissions at the farm level. This integration can help with proactive emission management, better decision-making, and effective mitigation strategy execution.

### 6.3. Policy Recommendations for Promoting Methane Reduction in the Livestock Industry

Effective policies are critical in encouraging methane reduction in the cattle industry. The following recommendations should be considered by policymakers:

Incentives and support: Financial incentives, grants, and subsidies should be implemented to encourage farmers to use methane reduction methods such as enhanced feed management, anaerobic digestion systems, and waste management technology. Farmers might benefit from assistance programs that provide technical expertise and financial resources to help them shift to more sustainable practices.

Education and training: Develop educational programs and training initiatives to enhance farmers’ understanding of the environmental impact of methane emissions and the potential advantages of mitigation techniques. Farmers can be empowered to undertake effective emission reduction measures if they have access to resources and training on best practices.

Research and development: Invest in research and development to advance livestock-specific methane reduction technology and techniques. Encourage researchers, industry stakeholders, and policymakers to collaborate in order to create creative solutions, enhance measurement techniques, and develop cost-effective mitigation plans.

International cooperation: Encourage worldwide cooperation and knowledge sharing to reduce global methane emissions from the cattle sector. Collaboration can promote the exchange of best practices, data, and technologies, resulting in more effective and coordinated methane emission reduction activities.

Emission reporting and verification: Establish strong reporting and verification processes to ensure accurate measurement, reporting, and verification of livestock methane emissions. Standardized methods and guidelines can improve the transparency, trustworthiness, and comparability of emission data, hence facilitating the achievement of emission reduction targets.

Governments may encourage sustainable practices, drive innovation, and accelerate methane reduction efforts in the cattle industry by implementing these policy proposals, thereby contributing to climate change mitigation goals.

## 7. Conclusions

### 7.1. Summary of Key Findings

In this review, we explored the significance of methane emission estimation within the cattle industry and its implications for climate change. Our investigation explains the various sources of methane emissions in cattle operations and underlines the critical need for precise assessment methods. We describe the techniques utilized in current methane measurement and implicated with the procedures for collecting methane emission data, encompassing both direct measurement techniques and indirect estimation approaches. Challenges, such as variability and data quality, were discussed in the context of methane data collection. Recognizing the limitations of traditional methods for estimating methane emissions, there is a growing acknowledgment of the role of AI technology in enhancing accuracy. This article elucidates a series of processes involved in utilizing AI technology for developing methane emissions estimation models for ruminants, spanning from data collection to processing and normalization. The review also investigated various models for estimating methane emissions, including VIF analysis, multiple regression, supervised learning models, and artificial neural networks (ANNs). These models exhibit higher accuracy and are better equipped to handle the complexities inherent in methane emissions within the livestock industry. The imperative for accurate methane measurement becomes apparent when evaluating the feasibility of achieving targets for inducing methane reduction from a policy perspective. This involves assessing the utility of feasibility assessments in incentivizing methane reduction and aligning with broader goals.

### 7.2. Potential Benefits of Accurate Methane Emission Estimation

There are various possible advantages to accurately estimating methane emissions. Firstly, it contributes to effective climate change mitigation efforts by providing critical data for understanding the environmental impact of the livestock industry. Policymakers can establish targeted policies and dedicate resources for emission reduction with accurate estimation. Second, it aids in the development of carbon trading systems by providing trustworthy data for the assessment of carbon credits. Accurate estimation contributes to the integrity and transparency of emission reduction activities, as well as the facilitation of equitable compensation for emission reductions.

### 7.3. Outlook for Future Research and Application

The continuing development and deployment of AI technology will determine the future of methane emission estimation. AI-based techniques have the potential to improve estimation model accuracy, scalability, and efficiency. Further research into the integration of AI with IoT for real-time monitoring and mitigation measures is required. Furthermore, advances in on-farm methane emission monitoring technology should prioritize portability, affordability, and ease of use.

Future study should also address the obstacles associated with methane data gathering, such as data variability, data quality assurance, and measurement technique standardization. Collaboration among researchers, industry stakeholders, and policymakers is critical for driving innovation, sharing best practices, and encouraging international collaboration in methane reduction efforts.

### 7.4. Summary

This article investigated the CH_4_ estimation in livestock, especially ruminants. Ruminants are one of the biggest anthropogenic sources of CH_4_ emissions in the world due to their special digestive system. With the worsening of global warming, humanity has agreed to reduce the amount of greenhouse gases emitted, and various policies for mitigation are being implemented. To ensure the implementation of policies for reduction of CH_4_ emissions from ruminants, it is essential to develop precise methods for CH_4_ measurement, but quantifying the CH_4_ emissions from ruminants always possesses uncertainty due to random effects. Employing inaccurate methods for measuring CH_4_ emissions from ruminants can lead to over- or underestimation in setting goals and implementing policies for methane reduction. Therefore, the development of precise methane measurement methods is crucial for achieving accurate targets and policy implementation. Advancing beyond conventional methods, incorporating AI technology in monitoring equipment and data collection procedures has the potential to significantly improve estimation accuracy. We may contribute to the worldwide effort to combat climate change and promote sustainable livestock production by incorporating accurate estimation into carbon credit evaluation and regulatory frameworks.

## Data Availability

The data presented in this study are available on request from the corresponding author. The data are not publicly available due to the availability of the data from the cited references.
